# Development of scaffold-free vascularized pancreatic beta-islets in vitro models by the anchoring of cell lines to a bioligand-functionalized gelatine substrate

**DOI:** 10.1007/s10856-022-06658-3

**Published:** 2022-04-11

**Authors:** Valeria Perugini, Samuel M. Flaherty, Matteo Santin

**Affiliations:** 1 Centre for Regenerative Medicine and Devices, School of Applied Sciences, University of Brighton, Huxley Building Lewes Road, Brighton, BN2 4GJ UK; 2 Division of Pharmacy and Optometry, School of Health Sciences, University of Manchester, Manchester, UK

## Abstract

Bioengineered pancreatic β-islets have been widely advocated for the research and treatment of diabetes by offering both suitable cell culture models for the study of the pathology and the testing of new drugs and a therapy in those patients no longer responding to insulin administration and as an alternative to the shortage of donors for organ and islet transplantation. Unlike most of the studies published so far where pancreatic islets of pancreatic β-cells are encapsulated in hydrogels, this study demonstrate the formation of bioengineered pancreatic islets through cell anchoring to a gelatine-based biomaterial, PhenoDrive-Y, able to mimic the basement membrane of tissues. Through simple culture conditions, PhenoDrive-Y led human pancreatic β-cell lines and human umbilical endothelial cell lines to form organized structures closely resembling the natural vascularized pancreatic islets. When compared to gelatine, the cultures in presence of PhenoDrive-Y show higher degree of organization in tissue-like structures, a more pronounced endothelial sprouting and higher expression of typical cell markers. Noticeably, when challenged by hyperglycaemic conditions, the cells embedded in the PhenoDrive-Y assembled spheroids responded with higher levels of insulin production. In conclusion, the present work demonstrates the potential of PhenoDrive-Y as substrate for the development of bioengineered vascularized pancreatic islets and to be particularly suitable as a model for in vitro studies and testing of new therapeutics.

**Figure:**
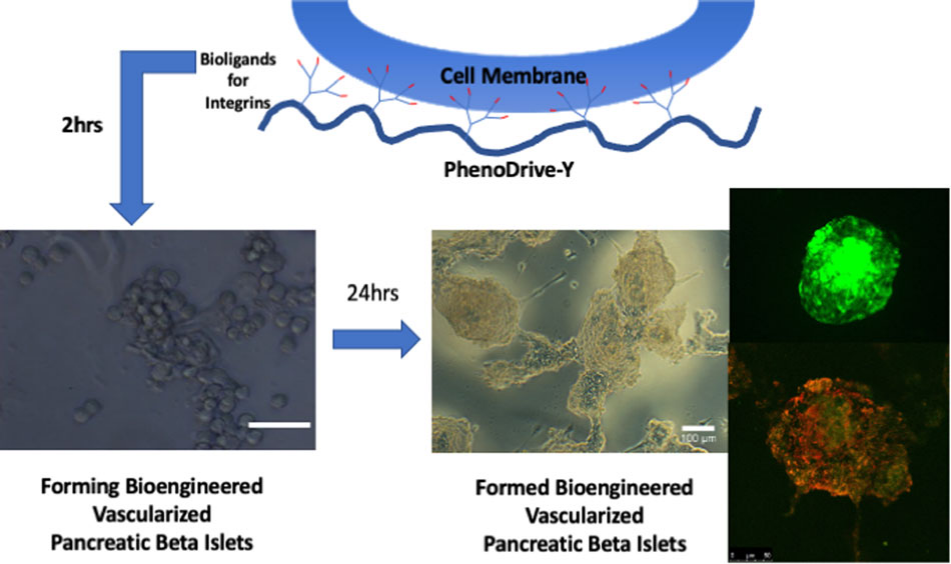
Graphical abstract

Graphical abstract

## Introduction

It has been widely recognized that the study of both type 1 and type 2 diabetes at a cellular level, as well as a future regenerative medicine treatment, strongly depends on the ability of developing bioengineered pancreatic islets. However, neither conventional tissue culture methods nor 3D cellular constructs can fulfil either the pre-clinical or the clinical needs as they fail to recapitulate the complex intracellular pathways and cellular signalling controlling the organ physiology or causing its pathological dysfunctions [1]. The use of isolated pancreatic islets is evidently restricted by the lack of sufficient numbers of donors and ethical issues related to their use for research rather than for transplantation.

The use of primary cells has been advocated in all these applications because their phenotype is preserved. However, the use of acinar, alpha-, beta-, ductal cells and pancreatic progenitor cells as well as pancreatic islets isolated from animal or human sources is restricted both by their availability, by their rapid de-differentiation when plated in 2D culture plates and by a short-term viability in 3D scaffolds or in suspension [2–4]. It has been long demonstrated that, because of their epithelial origin, the differentiated state of the pancreatic cells, particularly that of β-cells, depends on the presence of a microenvironment where their interactions with the basement membrane, as well as with other epithelial cells, are preserved [5, 6].

This observation has led to the gradual development of 3D systems where these types of interactions could be established. Early attempts comparing islet cell behaviour when plated on a collagen substrate or embedded into a collagen gel clearly demonstrated the ability of the cells to re-organize into islet-like structures in the 3D environment [7]. More recently, similar results have been obtained by encapsulating β-cells and pancreatic islets in hydrogels such as alginate, polyethylene glycol containing either laminin-derived cell adhesion peptides or the proteins laminin and collagen type IV of the basement membrane [8–10].

Pancreatic cell lines of animal and human origin have also been obtained and used in many studies [11]. However, it is widely accepted that these cell lines do not recapitulate the primary cell phenotype. Noticeably, many β-cell lines have a limited ability to produce insulin and, unlike their primary correspondent cell type, they have a higher rate of proliferation thus not adequately reflecting the primary cell cycle [1, 12]. While these limitations do not make cell lines suitable candidates for therapeutic applications, they can be considered valuable tools for in vitro study of pancreatic cell functions and testing of novel therapeutics. Among them, the 1.1B4 cell line, derived from the electrofusion of exocrine pancreatic cell line PANC-1 and freshly isolated human pancreatic β-cells, has emerged as a suitable in vitro model, but a limited ability to produce insulin has been observed up to 40 passages [12].

Progenitor cells have therefore been considered as an alternative to produce large batches of cells with primary phenotypes. Both human embryonic stem cells (hESC) and induced pluripotent stem cells (hiPSC) have been differentiated into pancreatic β-cells and used in the form of 3D aggregates resembling the pancreatic islets in mono-culture or in combination with endothelial cells to mimic the vascularised tissue [13–15]. Ethical issues concerning the use of hESC and the standardisation of the β-cell phenotype for iPSC have limited their use as a replacement of cadaveric pancreatic islet transplantation and as in vitro models for the study of diabetes and testing of new drugs [13].

The testing of new candidate therapeutics for the treatment of diabetes requires alternative in vitro models that can reduce animal experimentation and that are based on human cells organised in tissue-like structures allowing a rapid, reliable and cost-effective data throughput. These requirements have stimulated the development of organoids, typically defined as 3D cell aggregates designed to recapitulate in vitro the histology and function of the natural organ [16, 17]. Although the term organoid usually refers to models based on progenitor cells [1, 13, 14, 16–18], primary cells as those isolated from patients affected by cancer and cell lines have also been considered for their development [19, 20]. In addition, in vitro methods based on tissue culture plates or cell suspensions that are able to drive the formation of tissue-like structures without the need for cell encapsulation in biomaterials scaffolds or entrapment in hydrogels have been developed; the aim of these approaches was to eliminate any interference caused by the biomaterial physicochemical properties upon testing [1, 15, 21, 22].

In line with these approaches, the present paper shows a novel method of preparation of vascularized pancreatic islets in vitro based on the anchorage of the cells to a biomaterial mimicking the basement membrane of epithelia, PhenoDrive-Y [23–25]. Human cell line 1.1B4 were combined with human umbilical vascular endothelial cells (HUVEC) and cultured with PhenoDrive-Y in its gelatine formulation (gelatine-based PhenoDrive-Y) to present the laminin sequence YIGSR to the cells in a spaced manner [23]. Cell constructs closely mimicking the natural vascularized β-islets were obtained with very low concentrations of the biomaterial added to the cell suspension. Morphological and morphometric analysis and insulin production were evaluated alongside the expression of a typical pancreatic β-cell differentiation marker, the pancreatic and duodenal homeobox factor-1 (PDX-1) [26], and the endothelial cell marker CD31. Furthermore, the localization of gap junction protein such as Connexin-43, key to cell-to-cell communication [27], helped to identify pathways of connection occurring between the two types of cells in the final construct.

## Materials and methods

### Cell substrate preparation

Gelatine (Sigma, UK) and gelatine-based PhenoDrive-Y (Tissue Click, UK) were used as substrates to drive the aggregation of human β-cell lines and endothelial cells in suspension. Control gelatine and gelatine-based PhenoDrive Y were dissolved in serum-free cell culture media pre-equilibrated at 37 °C to obtain a 0.01% w/v solution immediately before the preparation of the 1.1B4/HUVEC cell suspensions. Preparations of the cell constructs were obtained in media suitable to both pancreatic β-cells (RPMI 1640 medium, Sigma, UK) and endothelial cells (F12K, Sigma UK).

### Cell cultures

Human pancreatic β-cells (1.1B4) were purchased from Sigma-Aldrich (UK) and cultured in RPMI 1640 medium containing 2 mM L-Glutamine and 10% v/v foetal bovine serum (FBS, Sigma-Aldrich UK). Whereas human umbilical endothelial cells (HUVECs) were grown in a complete cell medium previously prepared by adding 0.05 mg/mL endothelial cell growth factor (Sigma, UK), 0.1 mg/mL sodium heparin (Sigma, UK) and 10% v/v foetal bovine serum (FBS) to the F12K growth medium (Lonza, UK). Both cells were cultured under standard culture conditions (37 °C, 5%CO_2_) and used at passage lower than 25 (1.1B4 β-cells) and 32 (HUVECs), respectively.

### Vascularises pancreatic β-cell islet preparation

1.1B4 β-cells and HUVECs were resuspended at a total cell density of 10 × 10^4^/mL (6 × 10^4^/mL = 1.1B4 β-cells; 4 × 10^4^/mL HUVECs) in a serum-free RPMI/F12K medium (1:1 ratio) containing 0.01% w/v gelatine (Sigma, UK) or gelatine-based PhenoDrive-Y (Tissue Click, UK). The cell suspension was placed in 30 mL sterile tubes and left on a tissue culture rotator at 37 °C for 20 min before being transferred to 1 mL non-adherent petri dishes (NunC, UK). After 2 h incubation on an orbital shaker (80 rpm), cells were treated with 10% v/v FBS (Sigma-Aldrich, UK) and incubated for another 24 and 48 h under continuous, gentle agitation at 37 °C, 5% CO_2,_ 95% humidity sufficient to improve media flow around the formed 3D constructs without preventing their adhesion on the plate. To investigate cell response to glycaemia, the obtained vascularized pancreatic β-cell islets were also grown in RPMI/F12K medium supplemented with either 5 mM (normo-glycaemic medium) or 25 mM (hyperglycaemic medium) glucose and cultured in a cell incubator for 24 and 48 h.

### Light microscopy analysis of vascularized pancreatic ß-cell islet morphology

The formation of the cell constructs and final islets morphology were assessed by light microscopy at ×10 and ×20 magnification at each step of construct formation (2 h) and early incubation (24 h) by light microscope (×10 and ×20 objective lenses, Nikon Instruments, UK) and recorded using an image processing software (NIS-Elements, Nikon Instruments, UK). Construct size was measured as average of surface area (μm^2^) by ImageJ/FIJI analysis software using a cluster analyse plugin (https://imagej.net/plugins/cluster-analysis) from *n* = 6 confocal images, ×20 objective lens.

### Immunofluorescence staining

At 48 h, engineered islets were fixed with 4% v/v paraformaldehyde for 15 min at room temperature and rinsed three times in phosphate buffered saline (PBS, Sigma-Aldrich, UK). Nonspecific sites were then blocked with 5% w/v bovine serum albumin (BSA, Sigma-Aldrich, UK) containing 0.2% v/v Triton X in PBS for 1 h and stained with a primary antibody against CD31 (1:50, R&D system, UK), PDX-1 (1:10, Abcam, UK), insulin (1:50, Cell Signalling technology, UK) and Connexin 43 (CX43, 1:50, R&D system, UK). All primary antibodies were diluted in a 0.5% w/v BSA in PBS and incubated overnight at 4 °C. Following washings, islets were incubated in goat anti-mouse IgG secondary antibody conjugated to Alexa Fluor 488 and goat anti-rabbit IgG secondary antibody conjugated Alexa Fluor 555 (1:100, Fisher-Scientific, UK) for 1 h at room temperature, rinsed twice with PBS and counterstained with Hoechst 33342 (1 ug/mL, Cell Signalling technology, UK) for 5 min. Images were finally captured using both fluorescence microscopy with digital SLR camera (Nikon Eclipse TE2000-U) and a confocal scanning microscopy (Leica TCS-SP5).

Z-stack images of each 3D cell constructs were also taken and processed using Leica software (Leica Application Suite, LAS X).

### Insulin synthesis quantification

Insulin quantification was determined using ImageJ/FIJI analysis software (https://kpif.umbc.edu/image-processing-resources/imagej-fiji/) and expressed as corrected total cellular fluorescence (CTCF) that measured the normalised insulin fluorescence based on the below equation:

corrected total cell fluorescence (CTCF) = Integrated density − (Area of selected cell × Mean fluorescence of background readings).

Briefly, twenty random circular areas within the insulin immunofluorescence staining characterizing the highest intensity were quantified and 20 background readings were calculated for each culture type. Background readings were subtracted from the fluorescence intensity readings providing a true value of intensity. During all insulin fluorescence measurements, the area of analysis remained the same (pixels/1000 µm^2^).

### Statistical analysis

Data were expressed as mean ± standard deviation. Statistical calculations were performed using Minitab 17 (Minitab 17 Statistical Software, State College, PA). Analysis focused on a significant increase in β-cell cluster area cover and an increase in maximum insulin production by means of immunofluorescence intensity measurements when comparing modified gelatine culture substrate to the controls. One way ANOVA was applied with *p* < 0.05 taken to characterize a significant difference.

## Results

**Fig. 1:**
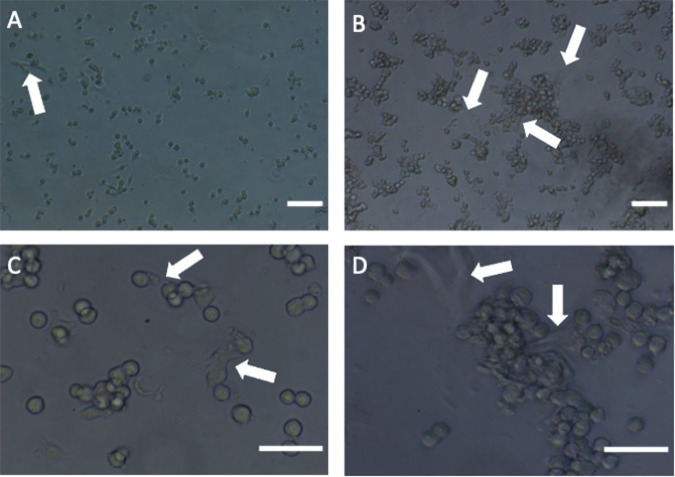
Morphological analysis of the forming human pancreatic β-1.1B4 cell lines and HUVEC constructs after 2 h incubation with gelatine (**A**, **C**) and gelatine-based PhenoDrive-Y (**B**–**D**). Arrows highlight forming endothelial sprouting. **A**, **B** = × 10 objective lens, **C**, **D** = ×20 objective lens

The early morphological analysis of the forming cell constructs after only 2 h of incubation on an orbital shaker clearly demonstrated that gelatine-based PhenoDrive-Y was able to accelerate the organisation of the cells in 3D spheroidal structures (Fig. 1A–D). Higher magnification also unveils the formation of early angiogenesis with tubuli-like structures being associated to the forming spheroids (Fig. 1A–D, arrows). These tubuli sprouting were not uniformly found in the gelatine constructs (Fig. 1A, C, arrows), while they were much more consolidated and integrated with the spheroidal structures when cells were co-cultured in presence of PhenoDrive-Y (Fig. 1B, D, arrows).

**Fig. 2:**
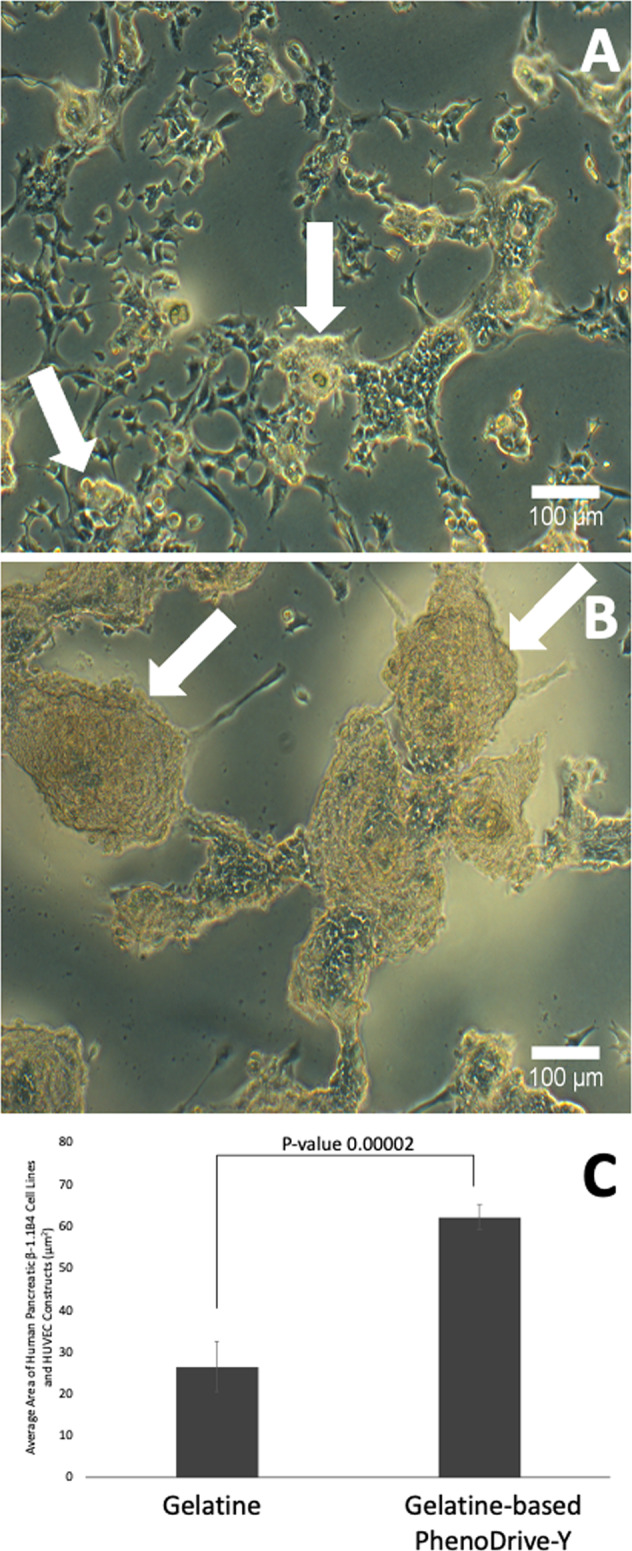
Morphological and morphometric analysis of the forming cell human pancreatic β-1.1B4 cell lines and HUVEC constructs after 24 h incubation with gelatine (**A**), gelatine-based PhenoDrive-Y (**B**), morphometry of engineered islets (**C**). Arrows highlight forming β-islet-like structures. Microscopy images were taken at ×20

The different cell organisation driven by gelatine and gelatine-based PhenoDrive-Y became more evident when constructs were analyzed by light microscopy after 24 h incubation (Fig. 2A, B). Both gelatine and gelatine-based PhenoDrive-Y were able to induce the formation of an endothelial sprouting network with 3D cell aggregates nesting at the anastomotic junctions (Fig. 2A, B, arrows), but the level of organization in larger structures resembling those of natural islets could clearly be observed in the case of co-culture with gelatine-based PhenoDrive-Y (Fig. 2B). Noticeably, islets formed by PhenoDrive-Y showed an area significantly larger than those induced by gelatine (Fig. 2C).

**Fig. 3:**
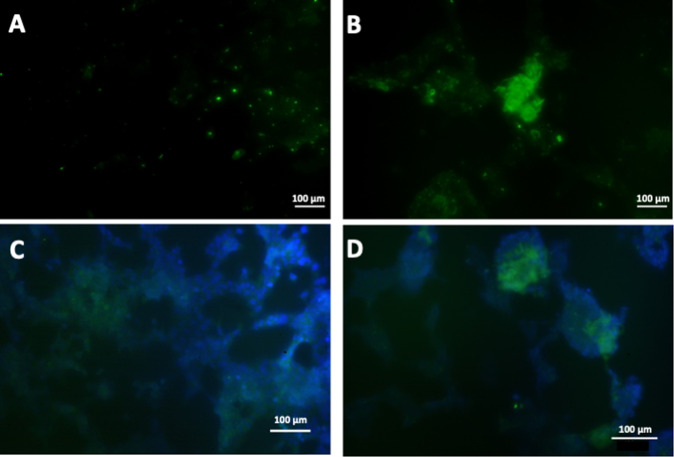
Immunostaining of the human pancreatic β-1.1B4 cell lines and HUVEC constructs for PDX-1^+^ human β-cells in co-culture obtained in presence of gelatine (**A**, **C**) and gelatine-based PhenoDrive-Y (**B**, **D**) at normo-glycaemic (**A**, **B**) and hyperglycaemic (**C**, **D**) conditions. Microscopy images were taken at ×10

Immunostaining for PDX-1^+^ human β-cells clearly confirmed the endothelial nature of the sprouting while showing the preferential localisation of β-cells at the anastomotic junctions (Fig. 3A, B). In the case of constructs obtained by incubation with gelatine, a limited expression of PDX-1 by the β-cells located at the anastomotic junction of the endothelial sprouting was observed (Fig. 3A), while a higher and very localised level of expression of this typical β-cell markers was found in the spheroids induced formed in the presence of gelatine-based PhenoDrive-Y (Fig. 3B). When the engineered pancreatic islet-like constructs driven by gelatine were incubated in hyperglycaemic conditions, a slight increase of the PDX-1 expression was observed (Fig. 3C), while the gelatine-based PhenoDrive-Y-driven constructs did not show any significant change in the expression of this marker (Fig. 3D).

**Fig. 4:**
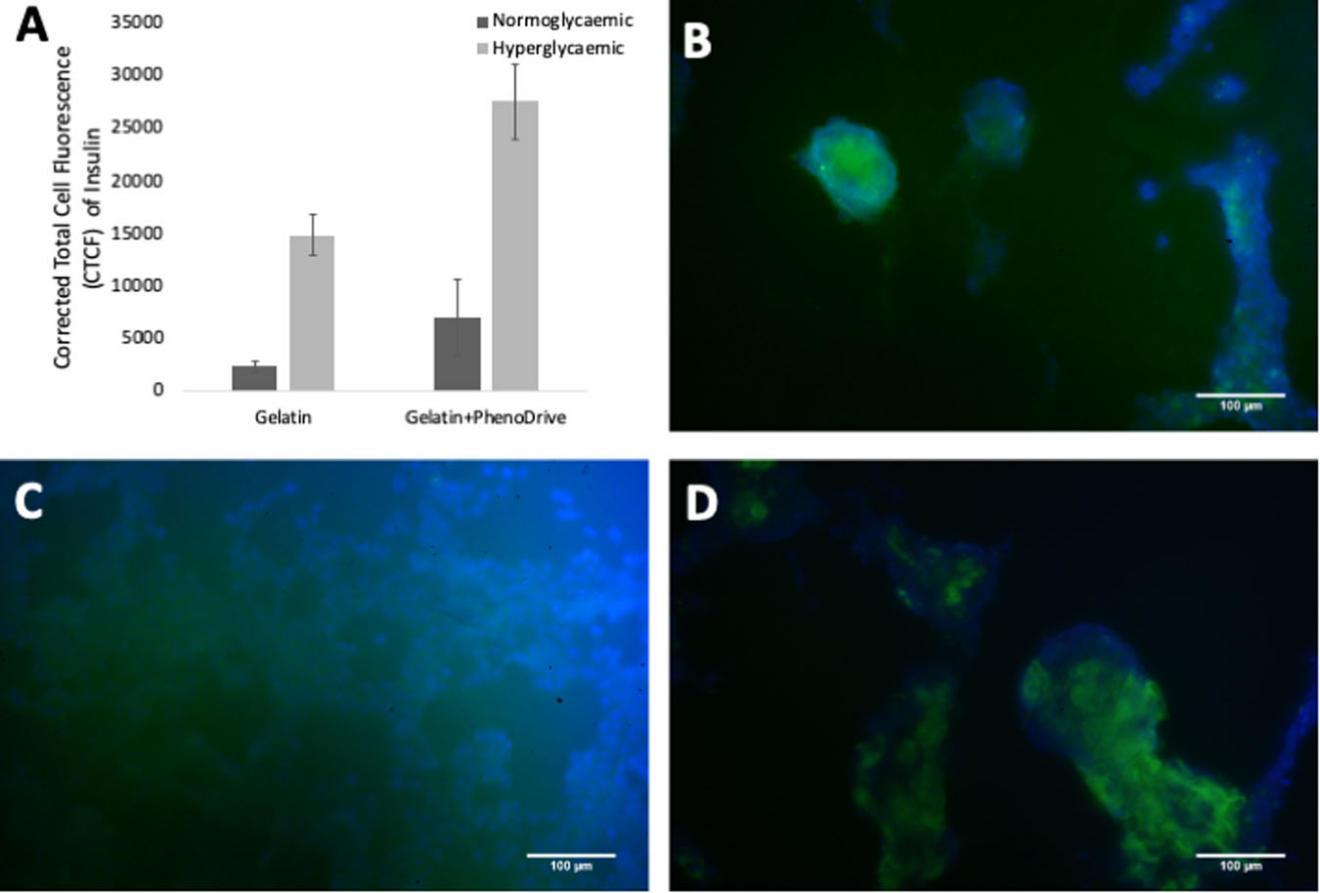
Immunostaining of the human pancreatic β-1.1B4 cell lines and HUVEC constructs for intracellular insulin synthesis when in normo-glycaemic and hyperglycaemic conditions. **A** Quantification of the immunostaining fluorescence, **C** gelatine constructs, **B**, **D** gelatine-based PhenoDrive-Y constructs. Microscopy images were taken at ×20 objective lens

The 1.1B4 cell line did not show any significant synthesis of intracellular insulin when incubated in normo-glycaemic condition (Fig. 4A) and only a slight diffused immunostaining could be observed after incubation in hyperglycaemic medium (Fig. 4B). A significant insulin synthesis was observed in the case of engineered β-islets obtained by PhenoDrive (Fig. 4A, C, D). More importantly, in these constructs the 1.1B4 cells responded to the incubation in hyperglycaemic medium by increasing the synthesis of insulin (Fig. 4A, D).

**Fig. 5:**
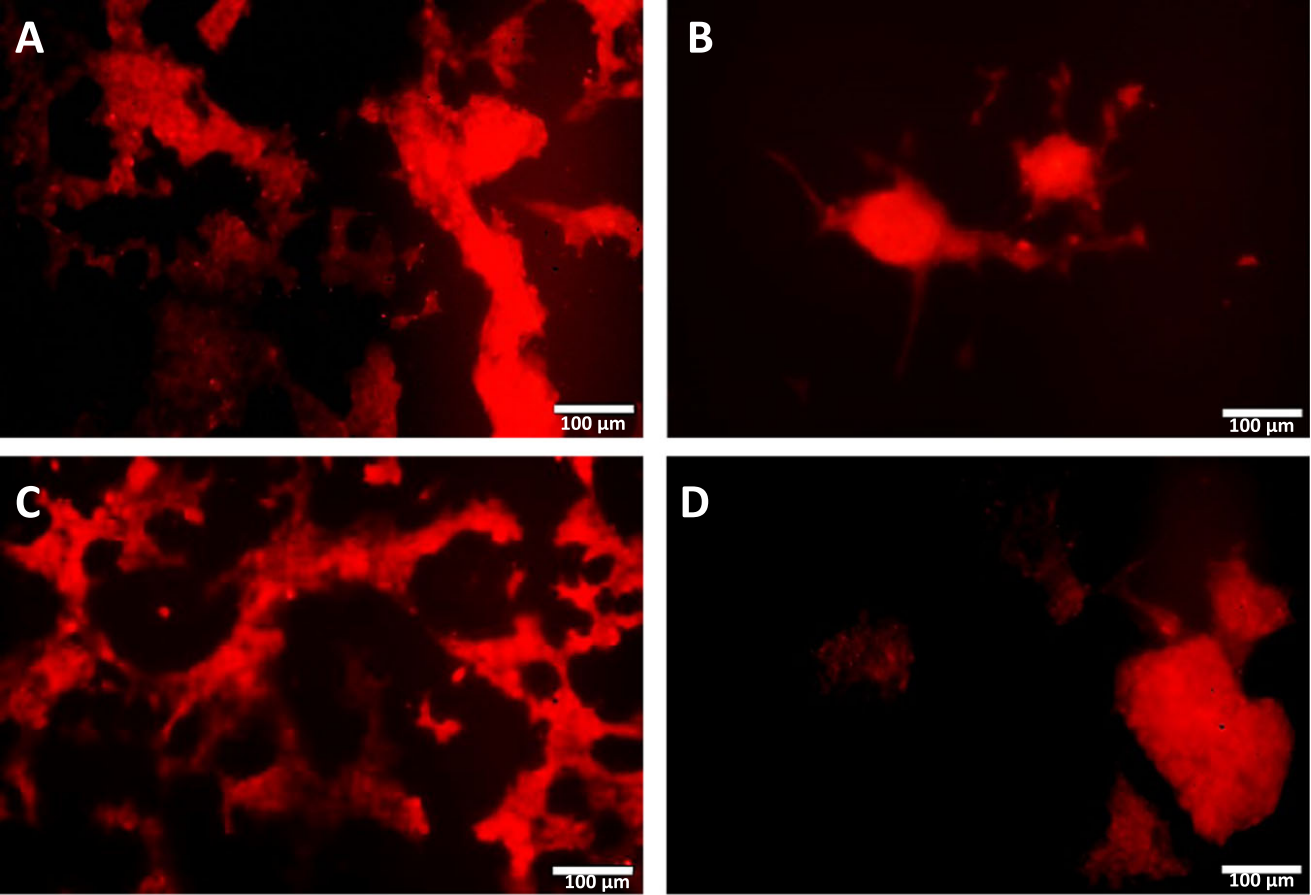
Immunostaining of the human pancreatic β-1.1B4 cell lines and HUVEC constructs for Connexin-43 expression in normo-glycaemic (**A**, **B**) and hyperglycaemic conditions (**C**, **D**). Gelatine constructs (**A**, **C**), gelatine-based PhenoDrive-Y constructs (**B**, **D**). Microscopy images were taken at ×20 objective lens

The study of the interactions between the β-cells and the endothelial cells in normo-glycaemic conditions indicated that, unlike the control gelatine where Connexin-43 was expressed rather homogeneously along the tubuli of the angiogenic sprouting (Fig. 5A), the engineered β-cells prepared by gelatine-based PhenoDrive-Y showed a preferential clustering of Connexin-43 at the anastomotic junctions of the angiogenic sprouting (Fig. 5B). When challenged by hyperglycaemic conditions, the Connexin-43 clustering in PhenoDrive-Y constructs appeared even more evident showing a tighter integration within the spheroidal cell constructs (Fig. 5C, D).

**Fig. 6:**
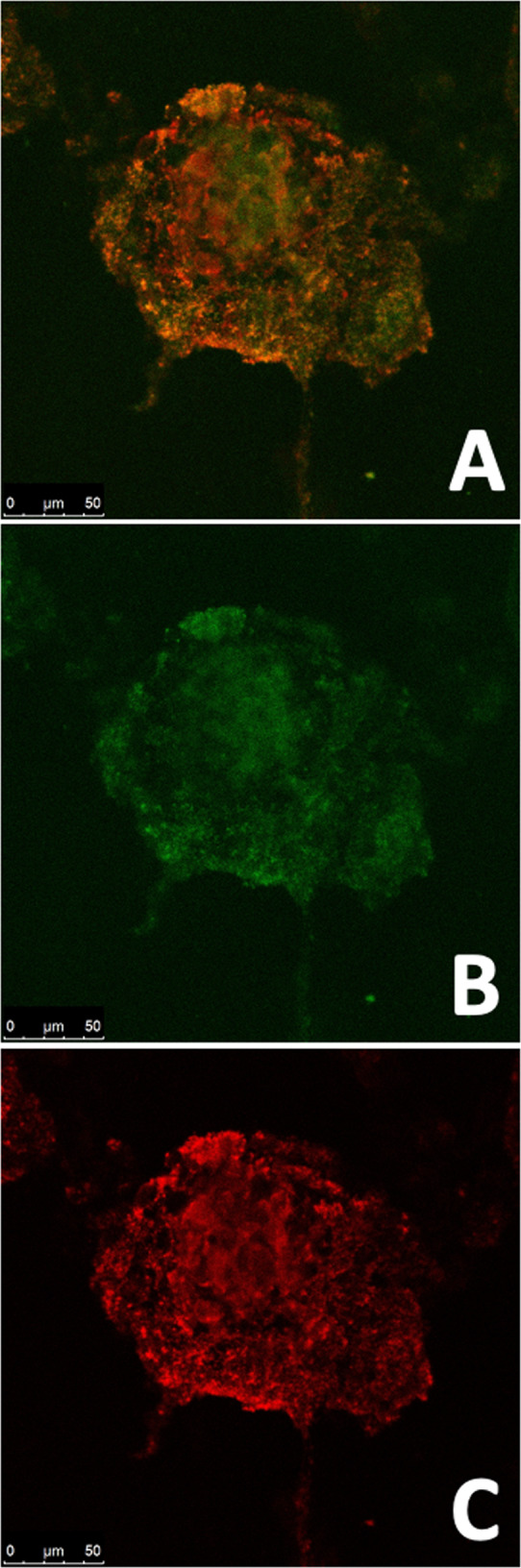
Confocal analysis of the human pancreatic β-1.1B4 cell lines and HUVEC constructs obtained by gelatine-based PhenoDrive-Y. Merged images of CD31^+^ endothelial cells and the PDX-1^+^ human β-cells (**A**) and single analysis of PDX-1^+^ (green-fluorescent staining, **B**) and CD31^+^ (red-fluorescent staining, **C**) cells. Microscopy images were taken at ×40 objective lens

**Fig. 7:**
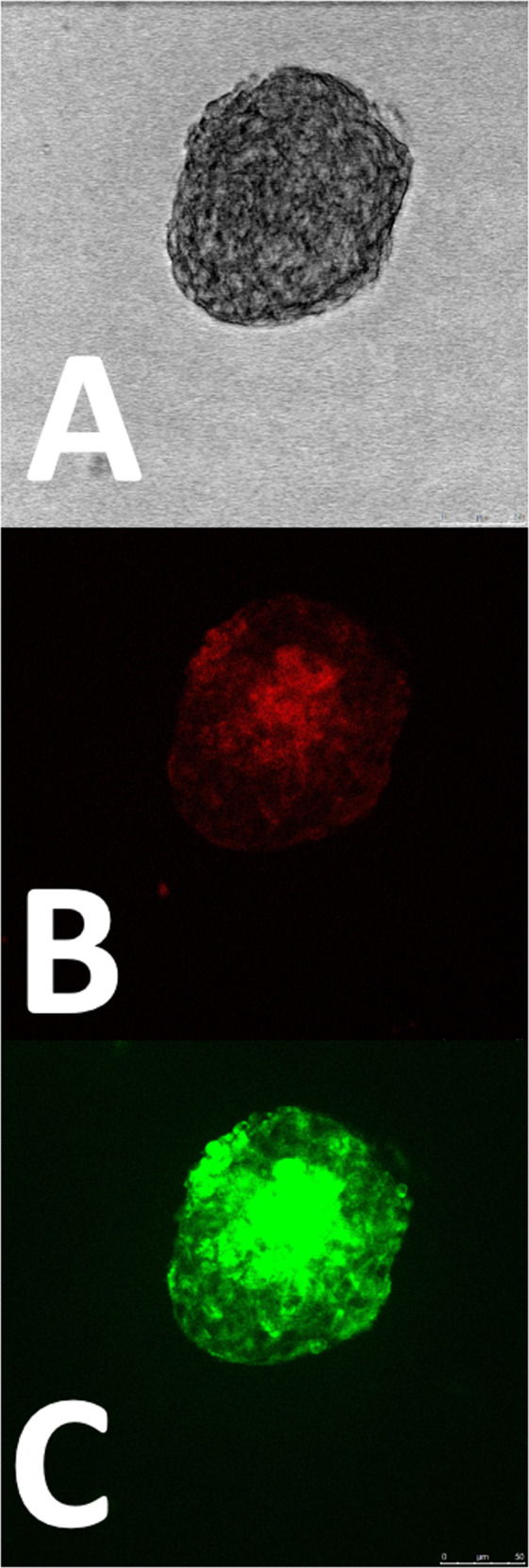
Z-stack confocal analysis of the human pancreatic β-1.1B4 cell lines and HUVEC constructs obtained by gelatine-based PhenoDrive-Y. Bright light microscopy (**A**) and single analysis of CD31^+^ (red-fluorescent staining, **B**) and PDX-1^+^ (green-fluorescent staining, **C**) cells. Microscopy images were taken at ×40 objective lens

Confocal analysis of the constructs obtained by PhenoDrive-Y provided a detailed analysis of the engineered islets showing the 3D structuring of the endothelial sprouting network with intercalated clusters of PDX-1^+^ cells (Fig. 6A–C). A denser core of this 3D spheroids was observed with a periphery characterised by a less dense mesh from which sprouting still emerged. This core was analyzed in more details by deconvoluting the whole spheroid (Fig. 7A) into z-stack images (Fig. 7B, C). This analysis showed the PDX-1 immunostaining following the same pattern of the intricate CD31^+^ angiogenic sprouting particularly visible at the periphery of the spheroid, further demonstrating the tight interactions between the two types of cells in the spheroidal structures.

## Discussion

There is a wide agreement for the need of in vitro pancreatic β-islet models suitable both to study cell interactions and signalling but also to test new drugs and drug carriers [28, 29]. Embryonic stem cells, as well as induced pluripotent stem cells have been used to develop organoids providing insights about pancreas development and pathological conditions [28, 30]. Likewise, it is known that the formation of cellular structures resembling those of the β-islets is not achievable in traditional 2D tissue culture plate where progenitor cells as well as isolated differentiated pancreatic acinar, alpha and β-cells lose their phenotype and that the encapsulation of these cells in hydrogels as well as genetic reprogramming are necessary [28, 31, 32]. Most of these studies have been focusing on the formation of pancreatic-like tissue structures starting from ductal cells tubule formations [31, 32] by the use of Matrigel, a collagen-based hydrogel containing a laminin fraction and widely used for cell encapsulation; this substrate has been shown to be able to induce cell organisation in a manner that recapitulates pancreas organogenesis [28, 31, 32]. These studies have provided evidence to show the importance of ductal epithelial cells to support the formation of pancreatic tissue-like structures in vitro and the important role played by the laminin in this process [28, 31]. However, it is argued that these models have not taken into account the vascularization of the endocrine β-cell islet histology and whether or not the endothelial cells of the microvasculature have a role in the organization of the β-cells. This role has been explored on animal cells alongside that of the extracellular matrix, particularly the basement membrane, in the formation of β-islets and their metabolism [33, 34]. Noticeably, both a physical and functional relationship has been demonstrated during the development of pancreas and still present in adults [35]. It has been shown that, within the pancreatic islets, β-cells have their apex oriented towards a capillary thus enhancing the exchange of signals and that mouse insulinoma cell lines are not able to synthesize a basement membrane unless surrounding the endothelial sprouting [35, 36].

Capitalizing on the availability of the gelatine-based PhenoDrive-Y, a substrate mimicking the basement membrane, this work demonstrated the close relationship between angiogenesis and pancreatic islet formation. The co-culture of HUVEC with the human 1.1B4 β-cells on this substrate showed the formation of organized structures in only few hours. Indeed, high magnification images clearly show the organization around the tubular endothelial cell sprouting (Fig. 1D). The data confirmed previous work showing the ability of gelatine-based PhenoDrive-Y to stimulate HUVEC sprouting and favour the interaction of the tubuli with human bone marrow stromal cells organised as spheroids at the anastomotic junctions [25]. Likewise, the morphological analysis after 24 h clearly showed that human β-cells seem to bind preferentially to the HUVEC angiogenic network at the anastomotic junctions. This preferential localization can be linked to the clustering of Connexin-43 in those areas of the angiogenic network (Fig. 5B, D). As time progresses, the size of the spheroids increased (Fig. 2C) and the tubuli-like structures became gradually encased in these 3D cell structures (Figs. 6 and 7). This was particularly evident when a z-stack of the cell spheroids is analyzed (Fig. 7B, C); the clustering of PDX-1^+^ cells being higher within the core of the spheroid (Fig. 7C), where the mesh of angiogenic sprouting of CD31^+^ cells was denser (Fig. 7B). The data collected over 48 h seems to suggest a mutual effect exerted by the two types of cells leading to the formation of β-islet-like structures. The formation of an early sprouting on a 2D plane seemed to be encouraged to be driven towards a 3D expansion by the β-cells sitting at the anastomotic junctions. Noticeably, the tight integration of the β-cells with the endothelial tubuli network led the 1.1B4 β-cells to express higher levels of their typical marker of differentiation, the PDX-1^+^ and to enhance their ability to respond to hyperglycaemic conditions by increased production of insulin. More importantly, this organotypic culture was obtained when cells were resuspended with very low concentrations of the PhenoDrive-Y thus reducing any significant effect of the biomaterial suggesting the potential of the developed organotypic culture as a suitable in vitro model for the study of pancreas regeneration as well as for the testing of therapeutic agents for endocrine pancreas diseases.
